# Recommendations for Infantile-Onset and Late-Onset Pompe Disease: An Iranian Consensus

**DOI:** 10.3389/fneur.2021.739931

**Published:** 2021-09-21

**Authors:** Farzad Fatehi, Mahmoud Reza Ashrafi, Marzieh Babaee, Behnaz Ansari, Mehran Beiraghi Toosi, Reza Boostani, Peyman Eshraghi, Atefeh Fakharian, Zahra Hadipour, Bahram Haghi Ashtiani, Hossein Moravej, Yalda Nilipour, Payam Sarraf, Keyhan Sayadpour Zanjani, Shahriar Nafissi

**Affiliations:** ^1^Department of Neurology, Neuromuscular Research Center, Shariati Hospital, Tehran University of Medical Sciences, Tehran, Iran; ^2^Children's Medical Center, Pediatrics Center of Excellence, Tehran University of Medical Sciences, Tehran, Iran; ^3^Physical Medicine and Rehabilitation Research Center, Shahid Beheshti University of Medical Sciences, Tehran, Iran; ^4^Isfahan Neurosciences Research Center, Alzahra Research Institute, Isfahan University of Medical Sciences, Isfahan, Iran; ^5^Faculty of Medicine, Mashhad University of Medical Sciences, Mashhad, Iran; ^6^Neurology Department, Mashhad University of Medical Sciences, Mashhad, Iran; ^7^Chronic Respiratory Diseases Research Center, National Research Institute of Tuberculosis and Lung Diseases (NRITLD), Shahid Beheshti University of Medical Sciences, Tehran, Iran; ^8^Medical Genetic Department, Atieh Hospital, Pars Hospital and Research Center, Tehran, Iran; ^9^Firoozgar Hospital, Iran University of Medical Sciences, Tehran, Iran; ^10^Neonatal Research Center, Shiraz University of Medical Sciences, Shiraz, Iran; ^11^Pediatric Pathology Research Center, Research Institute for Children's Health, Shahid Beheshti University of Medical Sciences, Tehran, Iran; ^12^Iranian Center of Neurological Research, Neuroscience Institute, Tehran University of Medical Sciences, Tehran, Iran

**Keywords:** Pompe disease, consensus, guideline, enzyme replacement therapy, Iran

## Abstract

**Background:** Pompe disease, also denoted as acid maltase or acid α-glucosidase deficiency or glycogen storage disease type II, is a rare, autosomal recessive lysosomal storage disorder. Several reports have previously described Pompe disease in Iran and considering increased awareness of related subspecialties and physicians, the disease's diagnosis is growing.

**Objective:** This guideline's main objective was to develop a national guideline for Pompe disease based on national and international evidence adapting with national necessities.

**Methods:** A group of expert clinicians with particular interests and experience in diagnosing and managing Pompe disease participated in developing this guideline. This group included adult neurologists, pediatric neurologists, pulmonologists, endocrinologists, cardiologists, pathologists, and physiatrists. After developing search terms, four authors performed an extensive literature review, including Embase, PubMed, and Google Scholar, from 1932 to current publications before the main meeting. Before the main consensus session, each panel member prepared an initial draft according to pertinent data in diagnosis and management and was presented in the panel discussion. Primary algorithms for the diagnosis and management of patients were prepared in the panel discussion. The prepared consensus was finalized after agreement and concordance between the panel members.

**Conclusion:** Herein, we attempted to develop a consensus based on Iran's local requirements. The authors hope that disseminating these consensuses will help healthcare professionals in Iran achieve the diagnosis, suitable treatment, and better follow-up of patients with infantile-onset Pompe disease and late-onset Pompe disease.

## Introduction

Pompe disease, also known as acid maltase deficiency or acid α-glucosidase (GAA) deficiency or glycogen storage disease type II, is an uncommon, autosomal recessive lysosomal storage disorder; it was initially described in a 7-month-old girl who deceased of cardiomyopathy (1).

The disease was recognized as a glycogen storage disorder wherein glycogen had accumulated within all the studied tissue vacuoles (1). The most acceptable classification of Pompe disease is based on the age of onset as the infantile-onset (classic and non-classic) and late-onset (childhood/juvenile and adult) forms. These forms vary according to clinical presentations, the organ involvement severity, and GAA activity levels.

The described frequency of infantile-onset Pompe disease (IOPD) ranges from roughly 1 in 35,000 in the Taiwanese population to 1 in 138,000 in Dutch people (2, 3).

In Iran, Pompe disease is mainly diagnosed and managed by neurologists (pediatrics and adult), pulmonologists, endocrinologists, physiatrist, and cardiologists (4–6). Several reports have previously described Pompe disease in Iran, and progressively, the disease's diagnosis is increasing, considering increased awareness of related subspecialties and physicians. This consensus's main objective was to develop a national consensus for Pompe disease based on national and international evidence adapting with national necessities.

### Method

Expert clinicians with specific experience and interests in diagnosing and managing Pompe disease participated in developing this consensus. This group included adult neurologists, pediatric neurologists, pulmonologists, endocrinologists, cardiologists, pathologists, and physiatrists.

The board was divided into two main groups: either IOPD or late-onset Pompe disease (LOPD). Initially, two main groups discussed previously defined subjects in separate groups and then joined together and finalized the main consensus. The main entities included: diagnosis of Pompe disease, treatment and management, rehabilitation and follow-up, and outcome measures.

After developing search terms, four authors carried out a broad literature review, including Embase, PubMed, and Google Scholar, from 1932 to current publications before the main meeting. The authors reviewed the abstracts of the related articles, and the publications were classified according to the level of evidence. The predefined headings and subheadings were distributed between panel members. Before the main consensus session, each panel member prepared a primary draft based on relevant data in diagnosis and management and was presented in the panel discussion.

A 2-day session was held, and the initial draft was discussed in detail. Every person was asked to contribute to the discussion by giving comments. Primary algorithms for the diagnosis and management of patients were prepared in the Panel discussion. The subject was voted and settled in case of disagreement when more than two-thirds of voters polled positively. After the panel, the discussions were continued in virtual group discussions made in WhatsApp messenger, and the prepared consensus was finalized after agreement and concordance between the panel members.

## Clinical Presentation and Diagnosis

Clinical manifestations of IOPD vs. LOPD are demonstrated in Table 1.

**Table 1:** Clinical manifestations of IOPD vs. LOPD.

**System**	**IOPD**	**LOPD**
Neurological/Musculoskeletal	Muscle weakness, motor delay developmental delay, absent or delayed motor milestones or even regression, hypotonia, poor head control, facial weakness with open mouth posture, tongue protrusion, weakness of proximal and truncal muscles, as well as distal muscles, calf hypertrophy, diminished reflexes, hearing loss	Preclinical hyperCkemia, exercise intolerance, cramps and skeletal pain, LGMD-like presentation, regional muscle involvement, paraspinal muscles involvement, waddling gait, difficulty in arising the chairs and going up the stairs, sensory neural hearing loss, aneurysmal arteries and secondary subarachnoid hemorrhage, cognitive impairment, like attentive and executive dysfunction, visuoconstructive impairment, painful paresthesia, autonomic dysfunction, rigid spine syndrome
Respiratory	Wet cough, respiratory distress, recurrent pneumonia, or upper respiratory infections, sleep apnea, respiratory failure	Respiratory insufficiency, daytime sleepiness, fatigue, and other symptoms related to chronic hypoxia and respiratory failure
Cardiac	Cardiomegaly, hypertrophic cardiomyopathy, congestive heart failure, rhythm disturbances such as supraventricular tachycardia	Arrhythmia, cardiac hypertrophy (less than IOPD)
Gastrointestinal	Hepatomegaly, feeding and swallowing difficulties, macroglossia, poor suck	Chronic diarrhea, abdominal pain, loss of appetite
Endocrine		Hypothyroidism

### Infantile-Onset Pompe Disease

As initially described by JC Pompe in 1932, the classic IOPD is characterized by rapidly progressive muscle weakness, primarily hypertrophic cardiomyopathy, feeding difficulties, and ensuing respiratory insufficiency. The symptoms are presented at roughly 3 months of age, and death happens at the age of 6–9 months, and around 10% of patients live older than 18 months. The onset of symptoms and cardiomegaly before 6 months are in favor of a poor prognosis. In the less common non-classic IOPD, manifesting in the first year of life, patients present with muscle weakness without cardiomegaly (2). These patients have a residual enzyme activity of below 20% (3). In the non-classic or milder form of IOPD, the onset of symptoms is much later (around half of these patients do not manifest until 4–11 months); these patients usually deteriorate more slowly than the classic IOPD patients (7).

### Main Clinical Presentations of Infantile-Onset Pompe Disease

#### Neurologic/Musculoskeletal

Patients with classic IOPD usually come to attention during their first 2 months of life with marked muscular hypotonia together with a rapidly progressing muscular weakness. Brain vasculopathy is mainly described in LOPD; however, patients with classic IOPD and brain arteriopathy are also described (8).

Muscle weakness and motor delay are the presenting manifestations in 40% of cases. The common neurologic/musculoskeletal manifestations include absent or delayed motor milestones and, in some patients, even deterioration, poor head control, hypotonia, facial weakness with open mouth posture and tongue protrusion, and generalized muscle weakness mostly involving proximal and truncal muscles; moreover, weakness of distal muscles, calf hypertrophy, and diminished reflexes may be observed (9).

Another clinical feature is hearing loss, recognized as an essential cause of morbidity in infants with classic IOPD (7). It stems from the accumulation of glycogen in the cells of the organ of Corti.

#### Respiratory

The usual respiratory symptoms consist of sleep apnea, cough, usually wet, respiratory distress, upper respiratory infections, recurrent pneumonia, and eventually, respiratory failure. Respiratory problems combined with recurrent respiratory infections are the first symptoms in higher than one-third of patients (10).

#### Cardiovascular

Congestive heart failure, cardiomegaly, hypertrophic cardiomyopathy, and rhythm disturbances such as supraventricular tachycardia are often present. The systolic dysfunction in IOPD usually occurs after 5 months of age (11).

#### Gastrointestinal

The main manifestations include hepatomegaly, feeding and swallowing difficulties, macroglossia, and poor suck failing to thrive. Hepatomegaly may stem from the background of congestive heart failure (12). Feeding difficulties are the first manifestation in half of the cases. Macroglossia is caused by the tongue's muscle fibers infiltrating with glycogen, but it is noted in less than half of patients. Detection of quantitative tongue atrophy by ultrasound may support the differentiation of LOPD from other acquired or hereditary myopathies (13).

### Late-Onset Pompe Disease

LOPD, in comparison to IOPD, has significant differences in presentation, severity, organ involvement, and disease progression. The most important and well-known clinical presentation of LOPD is skeletal muscle weakness and respiratory insufficiency. Still, this disease should be considered a multisystem involving disorder rather than a simple myopathy (14).

#### Neurologic/Musculoskeletal

Muscle involvement in LOPD presents as isolated hyperCKemia, exercise intolerance, mild to severe muscle weakness, and respiratory failure. Besides weakness, other muscular symptoms such as cramps and skeletal pain are also reported in LOPDs (15). The pattern of muscle involvement is also variable, from limb-girdle muscular dystrophy pattern to regional muscle involvement; lower limbs and paraspinal muscles are often involved much earlier than other parts, such as upper limbs and respiratory muscles. Muscles may be involved asymmetrically (16). In lower limbs, the involvement of gluteal muscles (hip extensors and abductors), adductor Magnus (thigh adductors), and Psoas (hip flexor) are more prominent than the other muscles, and this causes waddling gait, difficulty in rising from the chair, and climbing stairs (17).

Apart from muscular involvement, other systems are also involved in LOPD, such as the central nervous system, peripheral nervous system, endocrine, and cardiac. Sensory neural hearing loss is more prevalent in IOPD, but it may occur in LOPDs (18).

There is a risk of developing aneurysmal arteries and secondary subarachnoid hemorrhage in the brain, more prevalent in posterior circulation arteries (19). Other CNS manifestations include cognitive impairment, such as attentive and executive dysfunction, visuoconstructive impairment, and other cortical impairment disabilities (19). Painful paresthesia, numbness, and autonomic dysfunction, small fiber neuropathy, and peripheral nervous system involvement are described in LOPDs (20).

Severe involvement of paraspinal muscles, with structural changes in spinal skeletal bone, may cause rigid spine syndrome (21). Rigid spine syndrome is characterized by the limitations in the flexion of the cervical and thoracolumbar spine with multiple causes, including different muscle and skeletal disorders; LOPD should be considered in the differential diagnosis of the myopathic causes. Other muscles such as the scapular girdle, tongue, and bulbar and facial muscles may be involved less frequently (21).

#### Respiratory

Weakness of axial muscles is associated with early involvement of the respiratory muscles (intercostal muscles and diaphragm) and associated with signs and symptoms such as ineffective coughing, orthopnea, and exertional dyspnea, which are initial signs of respiratory failure (15). The respiratory consequences of muscle weakness, especially paraspinal muscles, result in restrictive lung disease, reducing vital capacity accompanied by a decrease in forced expiratory volume. Initial signs of respiratory distress present as diminished quality of sleep, daytime drowsiness, fatigue, headaches, and reduced pulmonary reserve. Weakness of respiratory and abdominal wall muscle induces ineffective cough and weakening of secretion clearance and airway protection (15). Other respiratory manifestations include morning headache, sleepiness, and sleep apnea.

A drop in postural forced vital capacity (FVC) (usually >25% decline from the sitting to the supine position) indicates reduced diaphragmatic strength. Increased inspiratory muscle weakness is assessed by maximum inspiratory pressure (MIP) and sniff nasal inspiratory pressure. Impaired coughing efficacy can be evaluated using maximum expiratory pressure (MEP) or peak cough flow (PCF). Weakness of respiratory muscle leads to sleep-disordered breathing and may progress to nighttime hypoventilation (15).

#### Cardiovascular

Cardiac abnormalities, including arrhythmia, cardiac hypertrophy, or Wolff–Parkinson–White and vascular abnormalities are notably less frequent in LOPD than IOPD (22).

#### Gastrointestinal

Gastrointestinal symptoms such as dysphagia, abdominal pain, chronic diarrhea, loss of appetite, malabsorption, and weight loss are reported in LOPDs; these improve by enzyme replacement therapy (ERT) (23).

#### Endocrine

Endocrine abnormality in LOPD is not prevalent, but hypothyroidism may be more frequent in these patients than in the general population (24).

### Investigations in Pompe Disease

#### Laboratory Investigations

##### Routine Blood Test

Creatine kinase (CK) rise is a sensitive, although non-specific indicator of Pompe disease. Around 95% of LOPD patients have raised CK. Increased CK can be found in some presymptomatic patients (25).

##### Tetrasaccharide in Urine

Glc4 is a sensitive, however nonspecific marker for Pompe disease (26, 27). Negative results from Dried blood spot (DBS) and Glc4 would exclude a diagnosis of IOPD, and it can be used as a biomarker (25).

#### Electrodiagnosis

It seems that in IOPD, the electrodiagnostic findings (nerve conduction study and electromyography) are time-dependent. Nerve conduction study is usually normal in these patients, and electromyographic findings range from myopathies with or without spontaneous activity to neurogenic changes (28); however, abnormal results are more common in LOPD. Such findings include spontaneous activity, such as myotonic discharges, in the absence of noticeable clinical myotonia (29). However, lack of myotonic discharges does not rule out the disease, and enzyme study is recommended for high clinical suspicion. Some adults suggested that myotonic discharge, especially in paraspinal muscles or tensor fasciae latae, is common despite normal findings in limb muscles, hence proposing the importance of paraspinal muscles assessment (28, 29). However, myotonic discharges and complex repetitive discharges of paraspinal muscles are not specific to Pompe disease and may be observed in some other muscle disorders (29).

#### Enzyme Assays

##### Dried Blood Spot

DBS is a rapid, reliable tool to measure the α-glucosidase activity in a dried blood spot. As a result, it is used as a screening test to assess patients with weakness of limb-girdle muscles or hyperCKemia (15). Its simple application has assisted in the early diagnosis of Pompe disease and its implementation at less advanced disease stages (30). As a screening tool, its variability in detecting LOPD in patients with unclassified limb-girdle muscular dystrophy phenotype or asymptomatic hyperCKemia ranges between 0 and 5% (31–33).

We recommend using DBS in patients with unclassified limb-girdle muscle weakness or asymptomatic hyperCKemia. DBS should be ordered at the initial stages of Pompe disease workup, particularly for an unexplained limb-girdle phenotype.

##### Leucocytes

GAA activity could be measured in mixed leucocytes from whole blood. The usage of acarbose to remove the interference by maltase glucoamylase improves the technique. Another possibility is to measure GAA activity in purified lymphocytes (2).

##### Fibroblasts

Another reliable method for GAA activity is measuring its activity in cultured skin fibroblasts. The limitation is that cultured fibroblasts need a skin biopsy that is a mini-invasive method. This measurement can take up to 1 month with a significant delay in the diagnosis. This test is used as a confirmatory assessment after a DBS positive test (15, 25); however, we recommend implementing genetic testing for Pompe disease following a positive DBS.

##### Blood Smear Examination

It has been demonstrated that assessing vacuolated lymphocytes in a blood smear is a potent marker in diagnosing autophagic myopathies (34). It can be considered a possible biomarker, and some authorities advise ordering blood smear examination to be performed even before GAA assay in DBS (35). Blood smear examination results need to be established by the finding of enzyme deficiency ± genetic analysis.

##### Muscle Magnetic Resonance Imaging

Muscle magnetic resonance imaging (MRI) findings in Pompe disease are consistent with spine extensors and pelvic girdle involvement and provide information on muscle changes, mainly in the tongue and subscapularis muscle (36). Also, it could be used as a biomarker for the patients' follow-up (37). It is shown that whole-body MRI may be beneficial in measuring muscle involvement in Pompe disease, especially at baseline, and recording disease progression in subjects treated with ERT (38).

#### Muscle Biopsy

Muscle biopsy is commonly used in the evaluation of neuromuscular patients. It can play a diagnostic or a supporting role in the diagnosis of Pompe disease (39). The procedure is minimally invasive. The muscle specimen must be directly frozen in isopentane, cooled in liquid nitrogen, and shipped on dry ice for histochemical enzyme and immunohistochemical studies.

**Figure 1:**
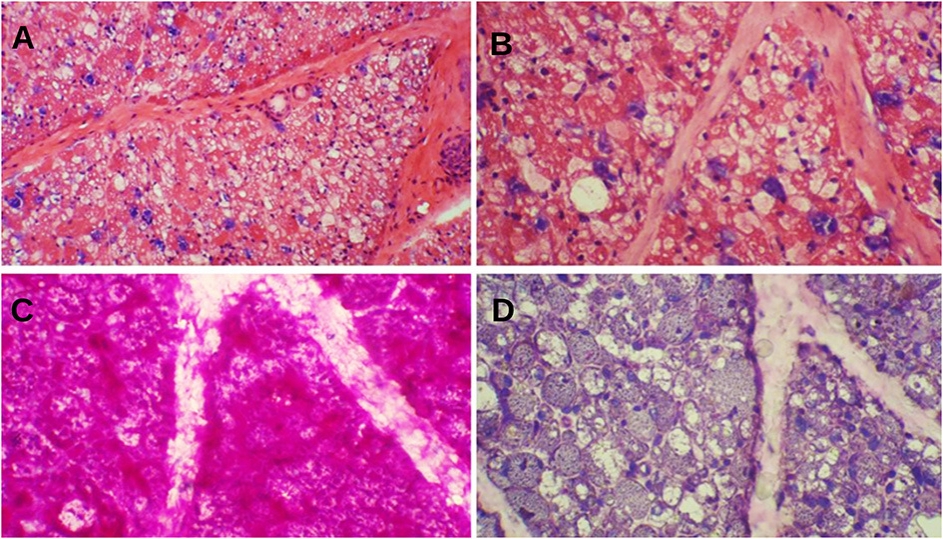
Muscle biopsy of a patient with Pompe disease. **(A)** Severe cytoplasmic vacuolization with presence of basophilic metachromatic materials (hematoxylin and eosin ×100); **(B)** Variable sized clear cytoplasmic vacuoles with granular basophilic materials (hematoxylin and eosin ×200); **(C)** Prominent glycogen excess [periodic acid–Schiff (PAS) ×200]; **(D)** Complete digestion of PAS + materials (PAS + diastasis ×200).

Muscle biopsy of classic IOPD reveals prominent vacuolar myopathy with fiber size variation. Many muscle fibers contain variable-sized round subsarcolemmal or cytoplasmic vacuoles. These vacuoles are either clear or include fine grayish materials, whereas some are filled by metachromatic debris, end products of autophagy. Periodic acid–Schiff stain shows prominent glycogen excess in almost all muscle fibers, although some vacuoles glycogen content is lost while processing the muscle specimens. Periodic acid–Schiff plus diastase stain reveals the digestion of all glycogen content (Figure 1). Acid phosphatase stain would show reactivity in cytoplasmic vacuoles and indicates their lysosomal origins.

#### Genetic Study, Consulting, and Prenatal Diagnosis

Pompe disease is inherited as an autosomal recessive disorder and requires both parents to be the carrier to develop the disease in offspring with a probability of 25% for each child (40).

The GAA gene is positioned in the human chromosome 17q25.2-25.3. It produces an inactive precursor transported to the lysosomal compartment and processed into intermediate and fully active forms (41). More than 580 different acid α-glucosidase gene mutations caused acid α-glucosidase deficiency and resulted in various clinical features, some of them causing a rapidly progressive disease (41). A common mutation in the Caucasian population is c.-32-13T>G splice mutation; it is found in IOPD and LOPD with an allelic frequency from 40 to near 100% in different studies (42, 43). All types of mutations are found in Pompe disease; the most frequent form is missense mutations observed in around half of the patients (42).

GAA analysis is suggested as a confirmatory test in the LOPD diagnostic algorithm. Approaches for identifying mutations include targeted panels, full sequence analysis, whole-exome sequencing, and deletion/duplication analysis (44).

After diagnosing Pompe disease, it is necessary to coordinate care by a team of expert clinicians to manage it (45). Genetic counseling is an imperative component of patients' care. It can be a source of the greatest-need essential information about the condition for patients' families (45). It is recommended to offer genetic counseling to all families with a diseased child and all adult-onset types through newborn screening, clinical diagnosis, or prenatal diagnosis.

Moreover, it is crucial to test siblings of an affected child for carrier detection (12). Carrier detection can be achieved by two foremost genetic approaches, biochemical testing or molecular testing. Pedigree with at least three-generation pedigree should be mapped for each family.

The physician can help the family comprehend the patient's situation, management, and treatment, the possible therapeutic options, including medications, rehabilitations, the expectations for the patient's response to the treatment, and the patient's probable prognosis. Parents of an affected patient should be obligatory carriers; the risk for affected offspring in these families in each pregnancy is 25%. DNA testing is necessary to identify the mutation in affected patients as the index case (12).

Prenatal diagnostic testing and carrier screening could be done by enzyme assay or mutation analysis (12). The prenatal diagnosis could be made by enzyme assay in amniocytes, but enzyme activity is usually lower than in chorionic villus samples (12). When the mutation in proband has been recognized, prenatal diagnostic testing by mutation analysis is more reliable and advantageous than biochemical methods (12).

## Differential Diagnosis of Pompe Disease

Rapid diagnosis of Pompe disease may be challenging due to variable clinical manifestations, wide-ranging phenotypic spectrum, the rarity of the condition, and clinical overlaps with other neuromuscular diseases. The differential diagnosis of Pompe disease is based mainly upon the age of onset of symptoms.

In classic IOPD, Danon disease, fatty acid oxidation disorders, mitochondrial disorders, spinal muscular atrophy type 1, congenital muscular dystrophies, and congenital myopathies are considered as differential diagnoses (12).

In LOPD, the vast differential diagnosis should be considered as limb-girdle muscular dystrophies, myotonic dystrophy type 2, facioscapulohumeral muscular dystrophy, Duchenne and Becker muscular dystrophies, congenital muscular dystrophies, myofibrillar myopathies, congenital myopathies, metabolic myopathies, mitochondrial myopathies, polymyositis with or without fibromyalgia, rigid spine syndrome, spinal muscular atrophies II and III, myasthenia gravis, and congenital myasthenic syndromes (46, 47).

## Diagnostic Algorithm

**Figure 2:**
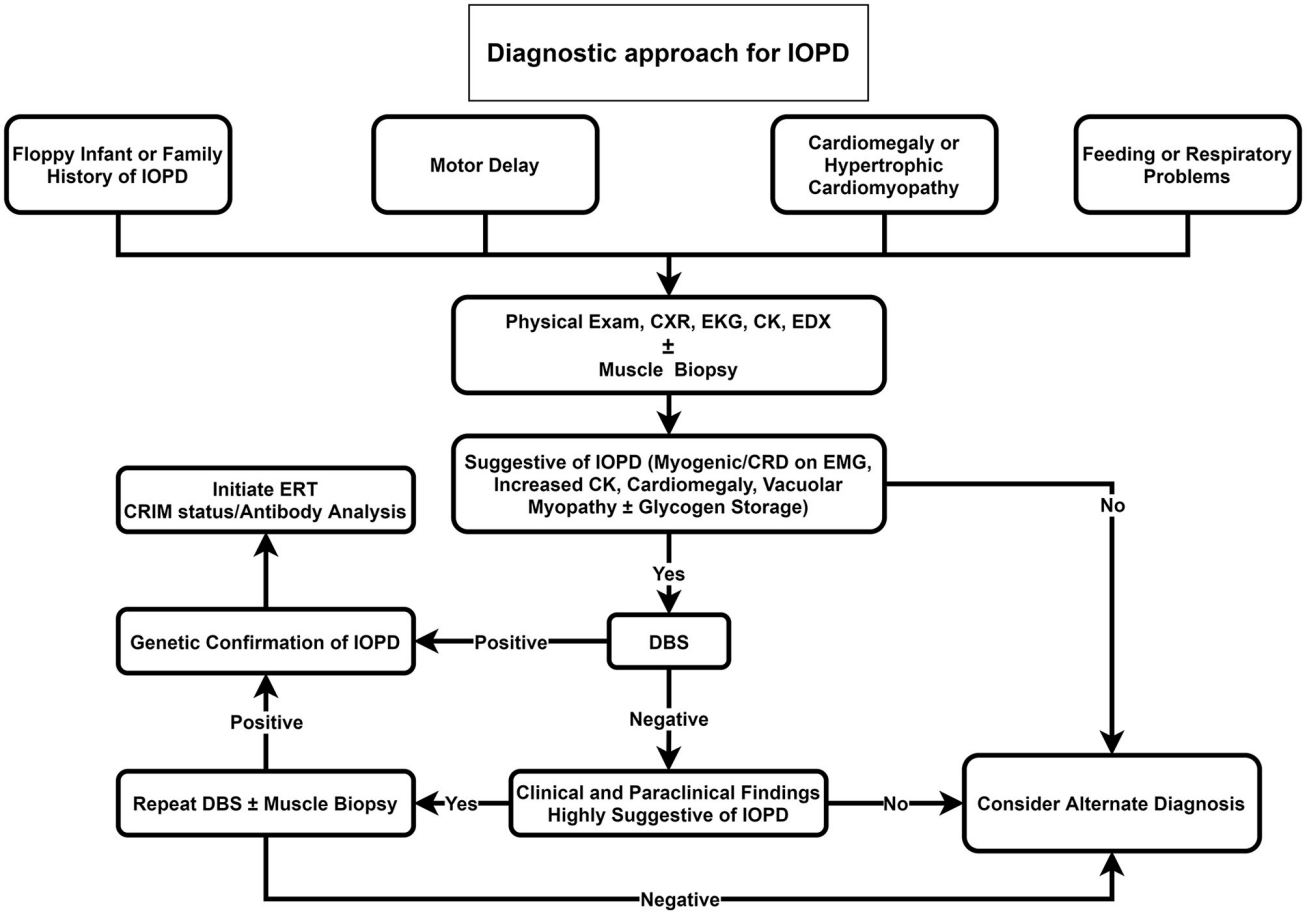
Diagnostic approach for IOPD. CK, Creatine kinase; CRD, complex repetitive discharge; CXR, chest X-ray; DBS, dried blood spot; EDX, electrodiagnosis; EKG, electrocardiogram; EMG; electromyography; ERT, enzyme replacement therapy; IOPD, infantile-onset Pompe disease; LOPD, late-onset Pompe disease.

**Figure 3:**
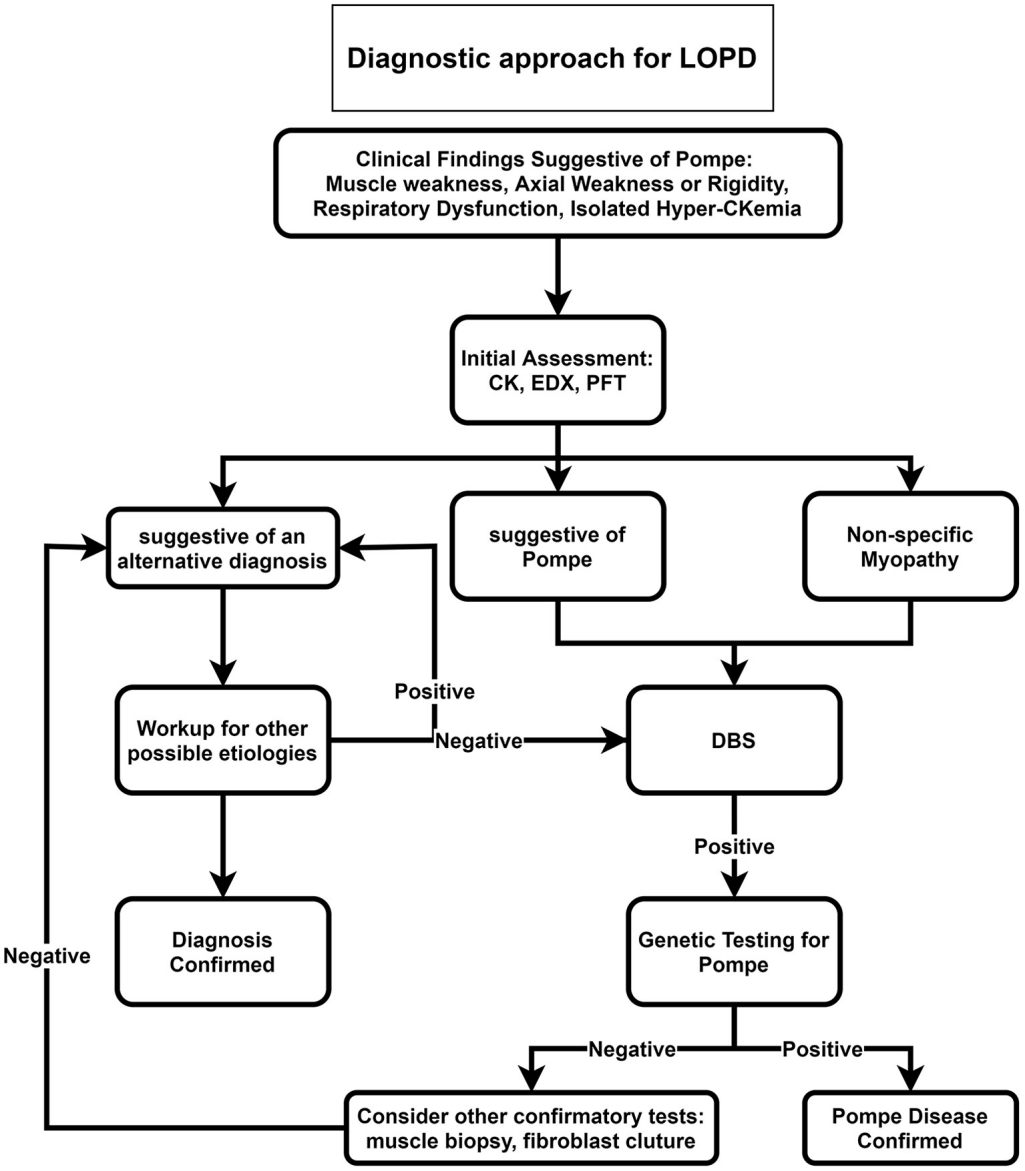
Diagnostic approach for LOPD. CK, Creatine kinase; DBS, dried blood spot; EDX, electrodiagnosis; LOPD, late-onset Pompe disease; PFT, pulmonary function test.

Our proposed diagnostic algorithms for IOPD and LOPD are depicted in Figures 2, 3, respectively.

We propose that DBS should be suggested as the initial investigation for all floppy infants or infants with a positive family history of Pompe disease, cardiomegaly, hypertrophic cardiomyopathy, feeding, or respiratory problems (Figure 2). If DBS was positive, in the next step, genetic testing for Pompe disease should be performed. Providing that DBS was negative, but clinical and paraclinical findings were highly suggestive of IOPD, we recommend repeating DBS or muscle biopsy if it was not performed before.

For late-onset patients, we suggest ordering DBS for all patients with muscle weakness, axial weakness, or rigidity, especially if accompanied by respiratory dysfunction or in the case of isolated hyperCKemia (Figure 3). After positive DBS, genetic testing is recommended, and if genetic testing was negative, confirmatory tests such as muscle biopsy or fibroblast culture are recommended.

## Outcome Measurements and Follow-Up

We divide the outcome measurement tools into respiratory and cardiac, motor function and quality of life, and blood tests.

### Respiratory Outcome Measurement

The diaphragm is prominently involved in Pompe disease, and its dysfunction causes significant respiratory symptoms (48). Pulmonary function tests (FVC, MIP, and MEP at the upright and supine positions) and polysomnography are suggested to assess these symptoms. A drop in postural FVC (usually >25% from the sitting to the supine position) indicates reduced diaphragmatic strength. Increased inspiratory muscle weakness could be detected by MIP and sniff nasal inspiratory pressure. On the other hand, MEP or PCF could detect impaired coughing efficiency.

### Motor Function and Quality of Life Outcome Measurement

The main motor function outcome measurements include manual muscle testing, quantitative muscle testing, a 6-min walk test, or a 10-m walk test. All reports in adults demonstrated that ERT could improve the walking and stabilization of respiratory function, especially in LOPD (49). Moreover, we suggest the Walton and Gardner-Medwin score and the Gait, Stair, Gowers' Maneuver, Chair group tests (G: gait by walking for 10 m, S: climbing four steps on a stair, G: Gower's maneuver, and C: rising from a Chair) that need only a few minutes to perform (50, 51). Quality of life measurement could be conducted by questionnaires such as SF36 for LOPD.

### Blood Tests

Blood tests include creatine kinase, alanine transaminase, aspartate transaminase, and antibody titers to anti-recombinant human GAA (rhGAA) immunoglobulin G (IgG).

## Treatment

### Infantile-Onset Pompe Disease

**Figure 4:**
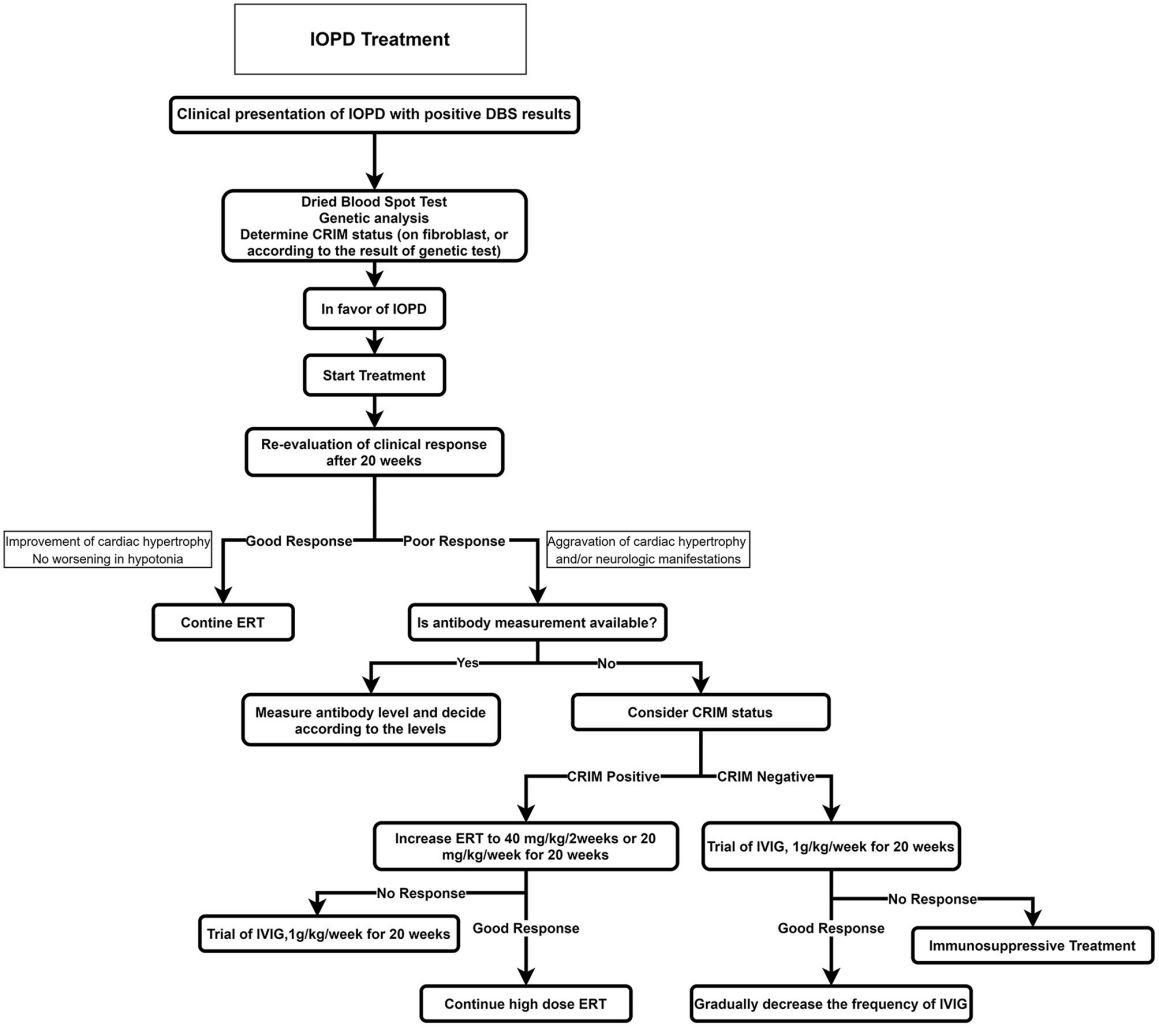
Treatment approach for IOPD. CRIM, cross-reactive immunologic material; ERT, enzyme replacement therapy; IOPD, infantile-onset Pompe disease.

The recommended algorithm for IOPD treatment is demonstrated in Figure 4. Treatment with alglucosidase alfa should be started immediately after positive DBS results or genetic confirmation. ERT may be started in patients with typical cardiorespiratory symptoms if the enzyme is deficient in DBS, even before seeing the genetic result (52). The usual recommended dose is 20 mg/kg alglucosidase alfa every other week; however, a recent study showed that patients treated with higher doses and more frequent injections had a better outcome such as motor, respiratory, and biochemical markers (53, 54).

Before starting ERT, a baseline evaluation of the cardiac, respiratory, and neurologic systems and developmental milestones should be recorded; cross-reactive immunologic material (CRIM) status should also be determined (if accessible). Providing that the CRIM test is not available, its status should be suggested according to the mutation. Alternatively, the anti-myozyme antibody should be measured, if accessible.

The first courses of ERT should be administered in an equipped pediatric center with excellent expertise on IOPD.

Severe life-threatening adverse effects such as cardiac arrest and cardiovascular collapse are more common in patients with an acute or severe disease than in other patients. Hence, ERT must be postponed in patients with acute infections, fever, or other critical illnesses. If the condition is continued, we recommend that ERT be done in an equipped center with an expert team for resuscitation (55).

### Cross-Reactive Immunologic Material Status and Immunomodulation

The patients are divided into two groups according to CRIM status: in cases with the complete absence of GAA by Western blot method, they are classified as CRIM-negative, and patients with detectable GAA protein are classified as CRIM-positive (56).

In general, most CRIM-negative patients on ERT have a worse prognosis because of emerging anti-rhGAA IgG antibodies (57). It is demonstrated that after 52 weeks on ERT, 5% of CRIM-positive IOPD patients died or were invasively ventilated compared to around half of CRIM-negative IOPD patients (58).

Some cases of CRIM-positive patients also build anti-rhGAA IgG antibodies such as CRIM-negative patients with an unfavorable prognosis. As a result, patients are classified into three subclasses according to anti-rhGAA IgG antibody titers (57): (1) High and sustained antibody titer, defined as antibody titer > 51,200 on samples at least 6 months on ERT; (2) sustained intermediate titer, defined as titers of ≥12,800 and <51,200 within 12 months on ERT; (3) low titer, defined as antibody titers of <12,800 during 1 year on ERT.

It is recommended to prescribe prophylactic immunomodulation to manage CRIM-negative status, including rituximab, methotrexate, rapamycin, mycophenolate, and intravenous immunoglobulins in various combinations (59). This approach has been more effective and with lower adverse effects in comparison with other therapeutic approaches. Recent studies also proposed novel immunomodulation agents that induce antigen-specific tolerance to ERT rather than systemic immunosuppressive agents (59).

The usually proposed regime includes rituximab 375 mg/m^2^ weekly for 4 weeks, followed by maintenance therapy, methotrexate 0.5 mg/ kg every week, and intravenous immune globulin 0.5 g/kg prescribed every 4 weeks (56).

### Late-Onset Pompe Disease

The most robust evidence for ERT benefits in LOPD originates from a randomized, placebo-controlled, prospective trial of rhGAA (60). In a systematic literature review of LOPD therapy (368 patients from 21 studies), as a minimum, two-thirds were stabilized or had improved CK levels and muscular ± respiratory functions (14). Also, a 5-year prospective study showed a long-term benefit of ERT in Pompe disease (61). ERT with alglucosidase alfa has increased life expectancy and survival in Pompe disease (62). A recent meta-analysis revealed that ERT might have a substantial favorable efficacy in the walking distance in LOPD patients; however, a non-significant amelioration of muscle power (63).

The delay between the first symptoms of the disease and ERT's beginning has shown an inverse relationship with the therapeutic prognosis and clinical outcomes desired (64).

There are two different commercially available rhGAA enzymes: Myozyme^®^ and Lumizyme^®^. Myozyme^®^ is produced by recombinant DNA technology in a Chinese hamster ovary cell line approved for all patients with Pompe disease in the United States and the European Union (65). Lumizyme^®^ is also produced by recombinant DNA technology but manufacturing on a larger scale, resulting in different product attributes. Currently, there is only one commercially available rhGAA in Iran, i.e., Myozyme.

The recommended dosage of Myozyme is 20 mg/kg bodyweight prescribed every 2 weeks through an intravenous infusion (66).

Incremental administration of infusions is recommended: the injection starts at an initial rate of 1 mg/kg/h and slowly increases by 2 mg/kg/h every 30 min if there are no signs of infusion associated reactions till a maximum rate of 7 mg/kg/h is reached (66).

The total time between reconstitution and completion of the infusion should not surpass 24 h.

Myozyme is well-tolerated, and the most adverse events are considered mild to moderate infusion-related reactions, including fever, urticaria, flushing, agitation, cough, nausea, and vomiting (60). All treatment-related adverse events are seen during infusion or within 2 h after infusion. Anaphylactic reactions are infrequent and are associated with a high titer of anti-rhGAA immunoglobulin E antibody. More infusion-related adverse events were reported for patients who received a higher dose of Myozyme (e.g., 40 mg/kg). Infusion-related adverse events might be managed by slowing or interrupting infusions, antihistamine, acetaminophen, and corticosteroids (60). ERT should be halted if the patient suffers from severe infusion-associated reactions that cannot be sufficiently managed (67).

### Recommendations

**Figure 5:**
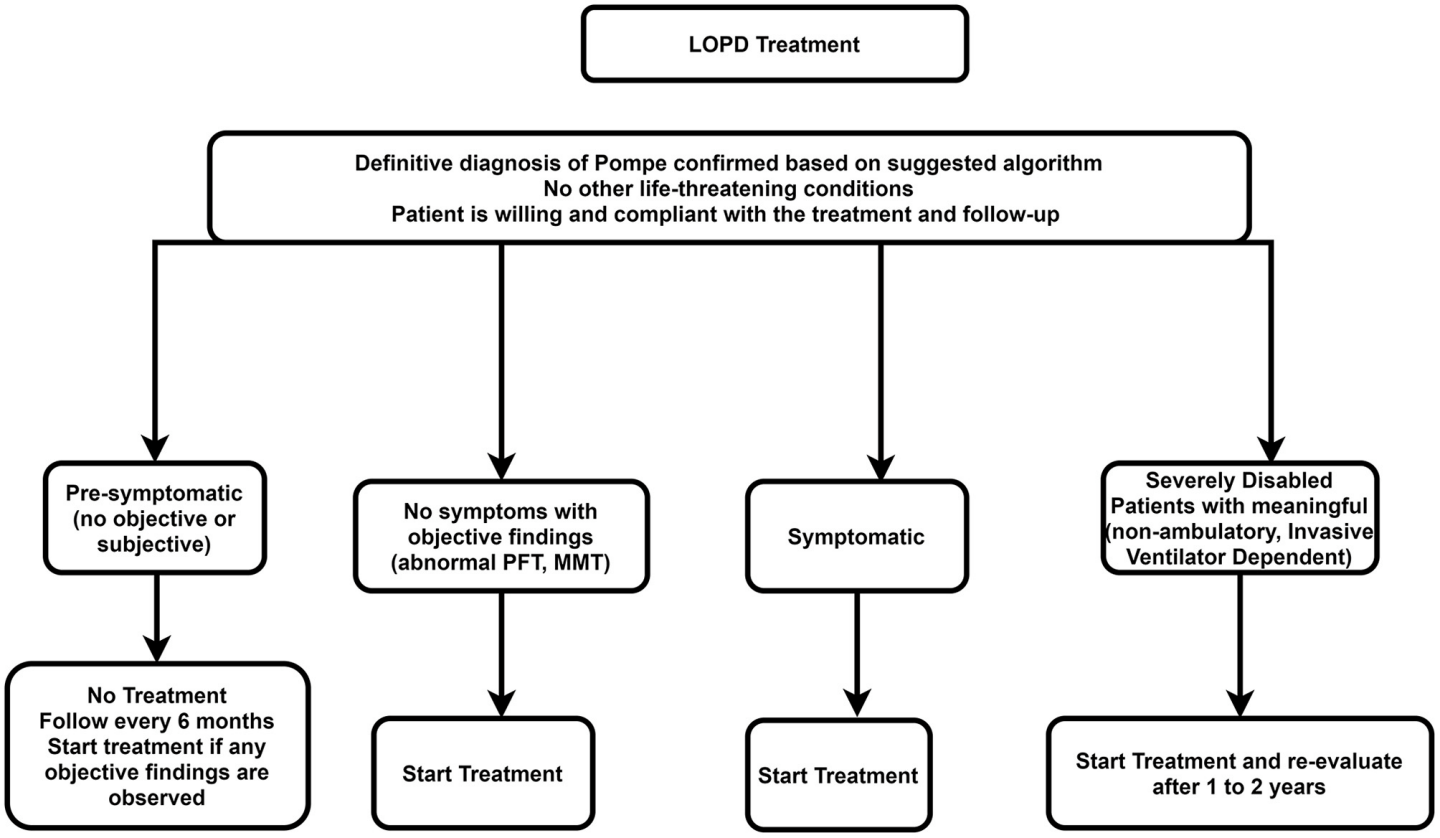
Treatment approach for LOPD. LOPD, late-onset Pompe disease; MMT, manual muscle testing; PFT, pulmonary function test.

The recommended algorithm for LOPD treatment is demonstrated in Figure 5.

#### Starting Enzyme Replacement Therapy

##### Presymptomatic Patients (Patients With No Weakness or Respiratory Involvement Whose Diagnosis of Pompe Disease Is Confirmed by Genetic Testing)

ERT is not necessary for presymptomatic patients. However, these patients should be checked every 6 months using the muscle strength test and pulmonary function test. Treatment with ERT should be initiated if there is evidence of weakness or impaired pulmonary function.

##### Symptomatic Patients

All symptomatic patients (i.e., patients with symptoms or signs of Pompe disease or impaired respiratory function) should start on rhGAA, depending on the patient's clinical status.

##### Advanced Stages of Disease (Non-ambulatory ± Receive Non-invasive Ventilation While Awake or Invasive Ventilation)

A trial of ERT may be considered in individuals with advanced stages of LOPD at a standard dose if there are predefined skeletal muscle outcomes, which can be assessed, if achieved, would improve the patient's function. In such cases, if the trial of therapy for 1–2 years does not deliver improvement in outcome measures, then the trial should be discontinued. However, if the disease deteriorates after stopping treatment faster than during treatment, restarting ERT can be an option.

#### Stopping Enzyme Replacement Therapy

rhGAA should be eliminated in the following situations: Severe infusion-associated reactions that cannot be effectively managed; the patient desires to stop ERT; lack of compliance of the patient with regular clinical assessments or infusions; accompanying another life-threatening disease. If no sign of improvement in skeletal muscle ± respiratory function was identified in the first 2 years after the start of treatment, the ERT should also be stopped.

It is recommended that multidisciplinary expert teams make decisions to stop the treatment to permit objective and careful consideration of all pertinent characteristics of the disease and its treatment, such as motor and respiratory functions and quality of life (68).

### Future Treatments

Developments in molecular medicine methods look promising for more effective therapeutic approaches. Approaches such as splicing modification by antisense oligonucleotides, stop codon read-through therapy, chaperone therapy, and the use of viral vectors (adeno-associated virus vectors) for gene therapy are encouraging therapeutic options in the future (69, 70).

## Rehabilitation

Rehabilitation should be considered the gold standard management in Pompe disease due to its nature, the involvement of several organs, and not complete remission after ERT (12, 66, 71, 72). Patients with Pompe disease, especially LOPD, are classified into presymptomatic, symptomatic, and severe based on the level of progression and disease severity (73, 74). Based on these classifications and complications (including respiratory insufficiency, cardiac, muscle weakness, scoliosis, nutritional, swallowing, and low bone mineral mass), the required rehabilitation will be determined (Table 2).

**Table 2:** Musculoskeletal, respiratory, and nutritional management of children with Pompe disease.

	**Stage 1**	**Stage 2**	**Stage 3**
Symptoms	Proximal muscle weakness or upright and supine FVC reduction.	Limb muscle weakness and upright and supine FVC reduction or difficulty in carrying out normal activities	Loss of ambulation
Musculoskeletal management (periodic review 3–6 months for children <5 years and annually for others)	– Evaluation of MMT, function (6MWT), and disability (PEDI, FIM), or ICF. – Evaluation of deformity: ROM, contracture – Evaluation of scoliosis and hip migration (in children) – Evaluation of scoliosis – DEXA for BMD – Pain evaluation (VAS) – Exercise: Mild to moderate intensity (60 – 70% of maximal effort); frequency of 3 – 5 treatment days per week – Stretching for lower limbs – Use of external device: AFO or TLSO		– Evaluation of MMT and disability (PEDI, FIM), or ICF. – Assessment of deformity: ROM, contracture – Evaluation of scoliosis and hip migration (in children) – DEXA for BMD – Pain evaluation (VAS) – Proper Positioning, active and passive ROM – Specific stretching for upper and lower limbs – Use of external device: AFO or TLSO
Respiratory management (periodic review 6 – 12 months) including FVC, the strength of the respiratory muscle (MIP and MEP), measurement of oxygen saturation (SaO_2_) at night, blood gas analysis, and transcutaneous monitoring of paO_2_ and paCO_2_	– Influenza and pneumococcal vaccination annually – Improvement/stabilization of vital capacity and respiratory muscle strength tests (MIP/MEP) by Respiratory muscle training (RMT) – Cough training or assistance	– Influenza and pneumococcal vaccination annually – Improvement/stabilization of vital capacity and respiratory muscle strength tests (MIP/MEP) by Respiratory muscle training (RMT) – Cough training or assistance – Increased PCEF (manual/mechanical support) – Sleep and life quality improvement by non-invasive ventilation [Continuous positive airway pressure (CPAP) or Bilevel inspiratory positive airway pressure (BiPAP)]	– Influenza and pneumococcal vaccination annually – Reduced ventilation hours (<8/day) – Change of the type of ventilation assistance (from controlled to assisted) – Cough training or assistance – Sleep and life quality improvement by invasive (Tracheostomy) or non-invasive ventilation – Tracheostomy removal
Nutritional	– Monitor growth parameters carefully – Provide adequate nutrition consisting of a high protein diet (20 – 25%) – Vitamins and minerals (including Ca, Vit D) – Swallowing maneuvers		– Consider the items in stage 1 – Swallowing maneuvers or posture – Videofluoroscopic swallowing assessment and evaluation for gastroesophageal reflux to guide management of feeding either orally or through a feeding tube – PEG is indicated for patients with severe dysphagia, aspiration risk, weight loss (>10% in 1 year), and FVC < 40%. – Evaluation for constipation

### Musculoskeletal Management

Motor and patient function assessment is recommended every 3–6 months early after diagnosis for children younger than 5 years and annually for children older than 5 years and adults (12, 74) (Table 2). A pulmonologist should visit these patients before exercise tolerance assessment due to their cardiopulmonary morbidity (73).

Exercise intolerance is mainly caused by muscle weakness rather than glycogenosis disorder (75, 76). Limited evidence is available for exercise therapy in these patients (75, 76). The exercise regimen of these patients should be initiated and extended gradually. Mild to moderate exercise (60–70% of maximal effort) is suggested 3–5 days a week (77). In this exercise regimen, stretching exercises should be added, and strenuous or eccentric physical therapy exercises, especially in proximal muscles, flexors, and abductors of the lower limbs, overwork weakness, and disuse atrophy should be avoided (72, 75–77).

After ERT, it seems that a combination of aerobic, resistance, and core stability has been beneficial and safe to ventilator-free patients who could walk alone and improved patient's pain, fatigue, and function (72).

### Low Bone Mineral Mass

It is suggested to perform an annual dual-energy X-ray absorptiometry bone mineral density assessment for LOPD child or adult wheelchair or ventilator-dependent patients (72, 78). Moreover, fall risk assessment is suggested for these patients due to high osteoporosis prevalence (71). If necessary, walker and cane use and instructions to prevent falling are advised and add calcium, vitamin D, and bisphosphonates to the patient's regimen (66, 77).

### Respiratory Rehabilitation

Based on the disease's course, a pulmonologist visit is suggested every 6–12 months, especially for children with LOPD, and pulmonary function should be assessed (Table 2) (72, 77). In non-cooperative children (older than 4–5 years), indirect tests such as measurement of oxygen saturation at night, blood gas analysis, and transcutaneous monitoring of partial pressure of oxygen and partial pressure of carbon dioxide are suggested (72, 77, 79). Blood gas and pulmonary function tests may be done annually or in case of a change in the patient's condition (77).

A drop in postural FVC (usually >25% from the sitting to the supine position) indicates reduced diaphragmatic strength. When PCF values decline below 270 L/min, use cough assistance such as manually assisted coughing, air stacking, insufflation/exsufflation, and high-frequency chest wall oscillation (66, 73, 80). Patients' nocturnal respiratory disorders may be managed by continuous positive airway pressure or bi-level inspiratory positive airway pressure.

### Nutritional and Gastrointestinal Evaluation

Pompe disease is presented with facial hypotonia, macroglossia, weakened tongue muscles, and oral movement impairment. The jaw muscles' fatigue can also cause an increased risk of aspiration and consume low amounts of vitamins, minerals, energy, malnutrition, and the compensatory use of muscle proteins. So speech therapy and swallowing techniques are recommended. Other gastrointestinal symptoms, such as dysphagia, gastroesophageal reflux, gastroparesis, and decreased bowel movement, are observed (12, 77). Videofluoroscopic swallowing assessment is recommended to assess patient condition, especially for aspiration risk (12, 66, 77).

However, the current ordinary care for Pompe disease is administering ERT; the treating physician should pay attention to the patient's exercise and nutrition. We suggest a high-protein and low-carbohydrate nutrition diet and exercise therapy (81). In patients with a motor disability that prevents regular daily activities, the total energy consumption by food must be reduced to evade obesity. Notably, lower total energy intake gives rise to a lower intake of protein and micronutrients. Pompe patients should be assessed regularly for nutritional deficiencies (protein, vitamin D, other vitamins) (51).

## Follow-Ups

### Infantile-Onset Pompe Disease

We recommend that the patients be visited by the physician every 3 months assessing feeding/swallowing, pulmonary and cardiac examination, and hearing status; however, the time interval may be modified based on the patient's clinical status (Table 3). A closer follow-up is required for patients with cardiac diseases. The physician should be attentive in testing for the hearing status associated with the disease intervening promptly if any hearing impairment is noticed (82). CK and liver function tests should be measured every 6–12 months.

**Table 3:** Recommended follow-up and assessment in classic infantile Pompe disease (CRIM-positive and CRIM-negative).

	**Assessment time point and frequency**				
	**Initial referral**	**2–4 weeks of age**	**Monthly to 4 months of age**	**Every 2 months (4–12 months of age)**	**Every 3–6 months (>12 months of age)**
**Clinical assessments**					
Feeding/swallowing	■		■	■	■
Chest X Ray	■				
Electrocardiography	■		■	■	■
Echocardiography	■		■	■	■
Holter cardiac monitoring	■		■	■	■
Auditory	■				
Developmental assessments	■	■	■	■	■
**Treatment evaluations**					
ERT antibodies (CRIM-negative)	■	■	■	■	■
ERT antibodies (CRIM-positive)	■	■	■	■	■
Pulmonary evaluation	■		■	■	■

### Late-Onset Pompe Disease

We recommend that the patients be visited by the physician every 6 months; however, the time interval may modify based on their clinical status.

**Figure 6:**
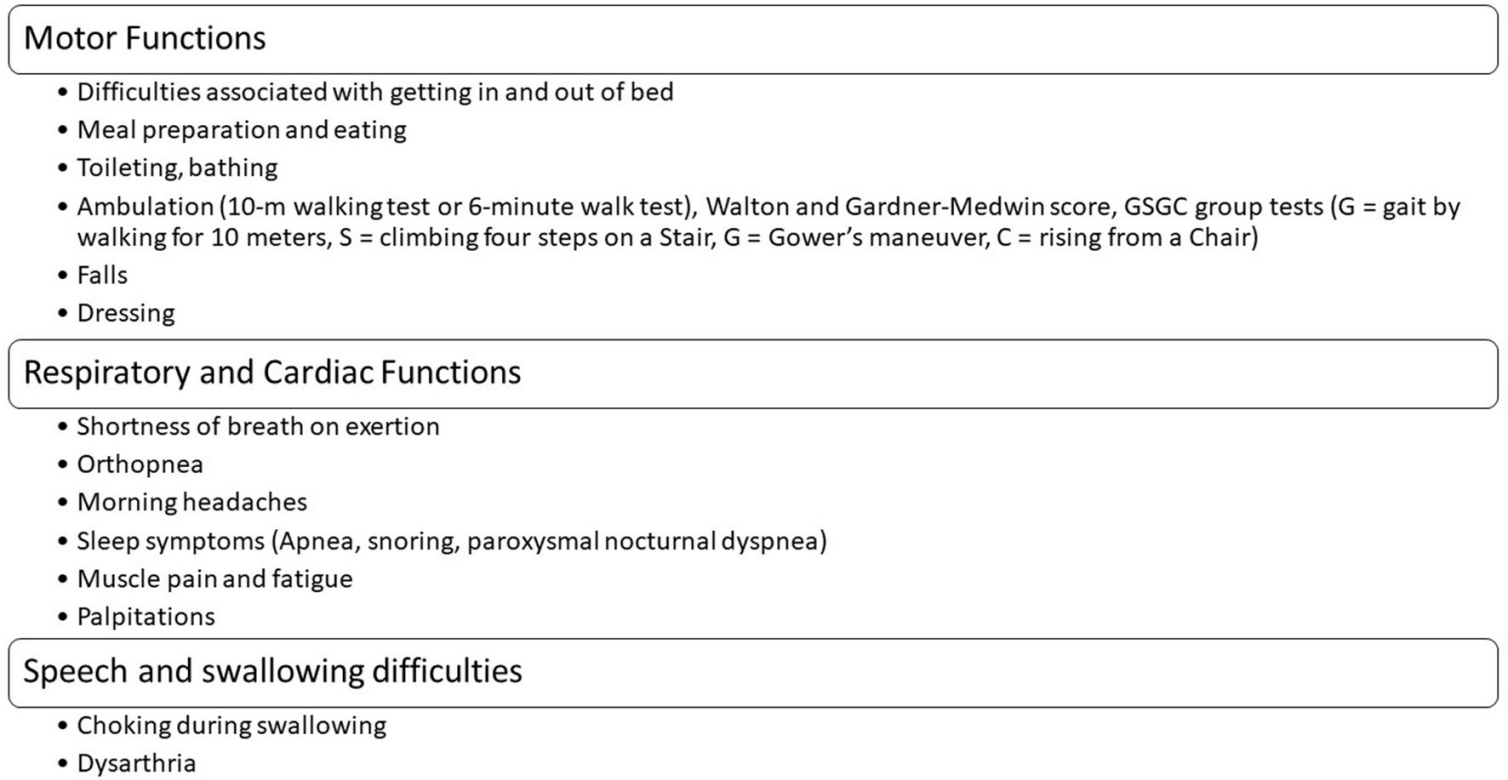
A minimum clinic visits inquiries for each session.

The following examinations should be performed in each session (Figure 6): Manual muscle testing, quantitative muscle testing, walking tests, spirometry, and evaluation for needing non-invasive ventilation or ventilator. CK and liver function tests should be measured every 6–12 months and respiratory function every 6–12 months, depending on the age of onset of patients' complaints. The following tests should be ordered at baseline and repeated regularly if clinically indicated: Brain MRI/magnetic resonance angiography, electrocardiography, echocardiography, bone mineral densitometry, and audiology assessment. Asymptomatic vertebral fractures without a history of trauma frequently occur in LOPD. Hence, screening for such fractures should be regularly accomplished regardless of the disease severity (83).

Gastrointestinal manifestations may cause a substantial disease burden on LOPD patients, and it is advised to be evaluated during clinical visits (84).

## Conclusion

Herein, we attempted to develop a consensus based on Iran's local requirements. The authors hope that disseminating these consensuses will help healthcare professionals in Iran achieve the diagnosis, suitable treatment, and proper follow-up of patients with IOPD and LOPD.

## Author Contributions

SN and FF organized the panel sessions. FF, MA, MB, BA, MBT, RB, PE, AF, ZH, BH, HM, YN, PS, KS, and SN contributed to consensus preparation, including literature review and writing the initial draft for each section, and also the final manuscript. All authors have read and approved the final manuscript.

## Conflict of Interest

The authors declare that the research was conducted in the absence of any commercial or financial relationships that could be construed as a potential conflict of interest.

## Publisher's Note

All claims expressed in this article are solely those of the authors and do not necessarily represent those of their affiliated organizations, or those of the publisher, the editors and the reviewers. Any product that may be evaluated in this article, or claim that may be made by its manufacturer, is not guaranteed or endorsed by the publisher.
